# Acute artery of Percheron stroke: To treat or retreat with thrombolysis?

**DOI:** 10.5339/qmj.2025.29

**Published:** 2025-03-18

**Authors:** Abeer Sabry Safan, Isra Eltazi, Khaled Zammar, Suhail Hussain, Ahmad Muhammad, Khawaja Haroon, Mostafa Mahmoud, Osman Koç

**Affiliations:** ^1^Department of Neurology, Neurosciences Institute, Hamad Medical Corporation, Doha, Qatar; ^2^Department of Interventional Radiology, Neurosciences Institute, Hamad Medical Corporation, Doha, Qatar *Correspondence: Abeer Sabry Safan. Email: asafan@hamad.qa

**Keywords:** Keywords: Bilateral thalamic strokes, artery of Percheron, ischemic, stroke

## Abstract

**Background:**

The artery of Percheron (AOP) stroke is a rare cause of bilateral thalamic strokes, which may or may not involve the midbrain. Existing literature has identified four anatomical variants of thalamic blood supply, with AOP being the IIB variant that arises as a solitary arterial trunk from either posterior communicating artery. The clinical manifestations of AOP strokes are diverse, with no specific localizing signs. Typically, patients present with symptoms such as amnesia, gaze palsy, and hypersomnolence. The predominant underlying etiology is often cardioembolic, requiring management strategies that are tailored to the source of emboli with anticoagulation/antiplatelets.

**Clinical presentation:**

We report a case involving a 72-year-old female patient with AOP stroke characterized by a sudden loss of vision, followed by a decreased level of consciousness. Magnetic resonance imaging revealed bilateral thalamic infarcts sparing the midbrain. CTA (computed tomography angiography) revealed a filling defect at the origin of the Percheron artery arising from the left P1 segment. The patient was treated with intravenous thrombolysis. The stroke workup was unremarkable, with a normal thrombophilia workup, a transthoracic echo, and no arrhythmias detected on a prolonged Holter monitor. The patient was treated with aspirin, atorvastatin, and intensive physical and cognitive therapy. On follow-up, she regained her consciousness but exhibited residual impaired vertical eye movements and right-sided dysmetria.

**Conclusions:**

AOP stroke is a radiological diagnosis with no specific localizing neurological signs. A high index of suspicion is essential for timely diagnosis and management, as bilateral thalamic involvement can arise from a wide range of metabolic, infectious, and other vascular etiologies that could delay optimal management.

## Introduction

In 1973, Gérard Percheron conducted a comprehensive examination of the blood supply to the thalamus, identifying the paramedian arteries as individual branches originating from the P1 segment of the posterior cerebral artery (PCA) that supplies ipsilaterally the paramedian thalami and occasionally the rostral midbrain.^1^ On rare occasions, paramedian artery variants may arise from a solitary arterial trunk that branches from the P1 segment on either side, which is known as the artery of Percheron (AOP).^2^ Ischemic infarcts involving the AOP often lead to bilateral thalamic strokes, with or without midbrain involvement, which can initially present as perplexing neurological constellations on a negative non-contrast computed tomography (CT).^2^ In the existing literature, the AOP (variant IIB) is recognized as one of four anatomical variants related to thalamic blood supply, as detailed in Table 1.^2^ The most prevalent pattern of ischemic AOP is characterized by bilateral paramedian thalamic with midbrain involvement (43%). Conversely, the least reported pattern involves the bilateral paramedian thalamic with the anterior thalamus sparing the midbrain (5%).^3^ The clinical presentation of AOP ischemic strokes typically manifests as amnesia, gaze palsy, encephalopathy, and hypersomnolence, which can be attributed to the involvement of the dorsolateral thalamus and the impaired function of the ascending reticular activating system (ARAS).^3,4^ AOP-associated ischemic infarcts are rare, representing approximately 0.4–0.5% of overall ischemic strokes, with a prevalence of 11% in the general population.^4^

In this report, we present a rare case of bilateral thalamic ischemic infarction resulting from an AOP variant, marked by the sudden onset of vision loss and impaired arousal. To our knowledge, the complete loss of vision has not been previously reported in the context of AOP strokes.

## Case Presentation

A 72-year-old right-handed, nonsmoker female with a medical history of diabetes mellitus, dyslipidemia, and hypertension was brought to the ED (emergency department) due to a sudden onset of bilateral vision loss, which was followed by a decreased level of consciousness. The collateral history was unremarkable for any previous headaches, constitutional symptoms, or similar episodes in the past, with no toxin exposure. The patient denied any recent travel that could suggest the possibility of paradoxical embolism resulting from deep vein thrombosis in the lower limbs due to long flights.

Upon her arrival at the hospital, an examination of her level of consciousness revealed flexion withdrawal to pain with incomprehensible sounds, as measured by the Glasgow Coma Scale (GCS E1V2 M4). Owing to her initial decreased state of consciousness, she underwent rapid sequence intubation. Therefore, the initial assessment of the National Institute stroke scale could not be performed accurately.

The patient's vital signs showed a temperature of 36.7°C, a heart rate of 67 bpm, a respiratory rate of 23 breaths/min, blood pressure of 120/72 mmHg, and an oxygen saturation of 100% while on mechanical ventilation. The cranial nerve examination revealed bilateral pupils measuring 3 mm, intact corneal and vestibulocochlear reflexes, and preserved cough and gag reflexes. There were no signs of pyramidal involvement. The initial CT of the brain did not reveal any intra or extra-axial bleeds, but it did show periventricular hypodensities that are suggestive of chronic microangiopathy. A CT angiogram of the head showed a filling defect at the origin of the Percheron artery from the left P1 segment. Clinically, a posterior circulation stroke was suspected due to a sudden onset of vision loss, followed by a decreased level of consciousness within 4.5-hour window for thrombolysis, with no known contraindication. Consequently, intravenous (IV) tissue plasminogen activator (tPA) was given with a bolus IV dose of 9 mg followed by an IV infusion of 81 mg over 60 minutes.

**Figure 1.**
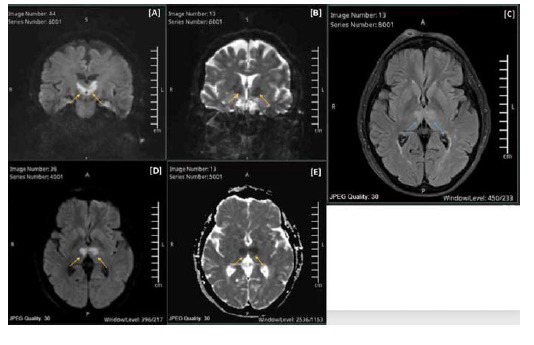
MRI head diffusion-weighted imaging (DWI). Images showing bilaterally symmetrical thalamic areas of diffusion restriction (yellow arrows) with corresponding high tbl2-weighted FLAIR signal intensity (blue arrows), which is suggestive of bilateral thalamic ischemic infarcts due to an artery of Percheron infarct.

The patient was admitted to the Critical Care Unit for intensive neuro-vital monitoring and supportive care. Magnetic resonance imaging (MRI) revealed bilateral thalamic areas of diffusion restriction on diffusion-weighted imaging (DWI), with corresponding high tbl2-fluid attenuated inversion recovery (FLAIR) signal intensity. These findings were suggestive of bilateral thalamic ischemic infarcts, most likely secondary to AOP occlusion (Figure 1). Consequently, the patient was diagnosed with AOP ischemic stroke.

Initial laboratory tests showed a normal CBC (complete blood count) and biochemistry with a random glucose level of 6.2 mmol/dL, hemoglobin A1c at 5.3%, LDL at 1.4 mmol/L, HDL at 1.3 mmol/L, cholesterol at 3.4 mmol/L, and a normal thyroid function test. Given the cardioembolic nature of AOP strokes, a comprehensive cardiac workup was conducted, which included an electrocardiogram that showed normal sinus rhythm and no abnormal rhythms or atrial fibrillation detected during 72-hour Holter monitoring. Further stroke workup with a transthoracic echo showed mild concentric left ventricular hypertrophy and severely dilated left atrium end-systolic volume index (57.6 ml/m^2^). The ejection fraction was measured at 61%, with no indications of left ventricular thrombus or patent foramen ovale.

Subsequent follow-up CT of the head, conducted after thrombolysis, showed bilateral thalamic hypodensities with no evidence of reperfusion hemorrhage. Therefore, the patient was started on aspirin (ASA) 100 mg and atorvastatin 40 mg daily. Her management plan involved a multidisciplinary approach, during which sedation was gradually reduced. On day six post-tPA, the patient was fit for extubation, with an overall GCS score of 10/15, spontaneous eye-opening, and the ability to follow simple commands. Neurological assessment showed impaired vertical eye movements bilaterally with right-sided dysmetria.

The patient trial of ORG 10172 in Acute Stroke Treatment Classification was labeled as an embolic stroke of unknown source^5^. She was transferred to a rehabilitation institute for intensive physical and cognitive therapy aimed at secondary prevention of stroke (ASA 100 mg daily orally and atorvastatin 40 mg daily bedtime) with a scheduled prolonged Holter monitoring. A stroke clinic follow-up revealed remarkable recovery, although the patient exhibited residual impaired vertical eye movements and right-sided dysmetria.

## Discussion

**Figure 2.**
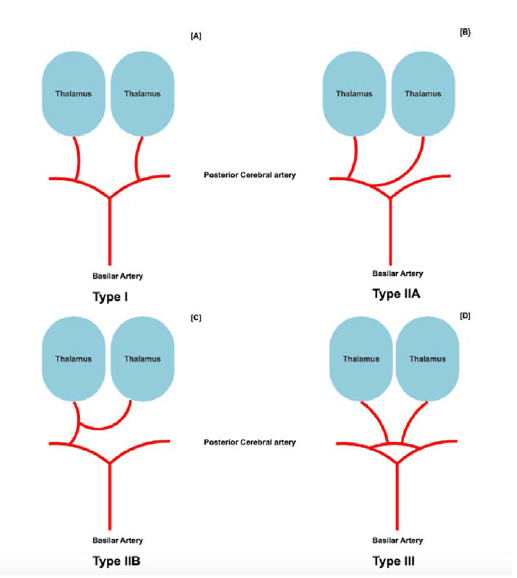
Variants of thalamic arterial supply (IIB – artery of Percheron).

The literature indicates that thalamic arterial supply is variable, with four discernable variants identified (Figure 2).^6^ The most common variant is variant I (tuberothalamic territory), with perforating arteries on each side arising from the respective right and left posterior communicating arteries (PCA).^6^ Vascular compromise may manifest as memory impairment, executive and learning dysfunction, and impaired arousal.^6^ The inferolateral territory corresponds to variant IIA, with the proximal segment of one PCA providing a perforating artery in an asymmetrical manner (Figure 2). Ischemic stroke in this territory could lead to contralateral hemisensory loss, hemiparesis, hemi-ataxia, and the sequela of post-stroke pain syndromes, which are predominantly observed in cases involving the right side.^2,6^

AOP is variant IIB, known as the choroidal territory, with bilateral perforating arteries arising from the solitary trunk from either PCA. Commonly reported sequelae include visual field deficits, extrapyramidal signs, hypersomnolence, and coma with poor outcomes.^6^ Finally, the paramedian territory (variant III) consists of several small perforating branches arising from one artery, serving as a bridge between P1 segments of bilateral posterior communicating arteries.^6^

**Figure 3.**
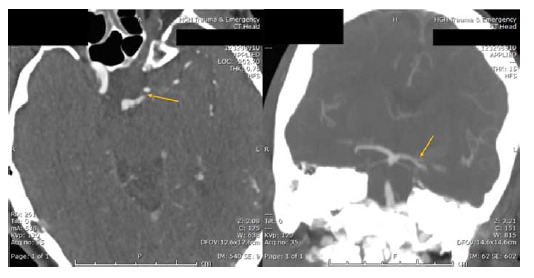
Computed tomography angiography of the head. There is a filling defect noted at the origin of the Percheron artery from the left P1 segment. This defect is clearly visible in both coronal and axial cuts (yellow arrows).

AOP is considered a radiological diagnosis, with brain MRI being the optimal modality due to its nearly 100% sensitivity. In contrast, CT of the head often misinterprets bi-thalamic abnormalities (sensitivity 55%), especially in early presentations.^7^ The possibility of an AOP variant infarct must be considered in the absence of basilar tip occlusion.^8^ Early diagnosis is best achieved through DWI, which reveals characteristic diffusion restrictions in bilateral thalami, as observed in our patient.^7^ However, a co-founding characteristic known as the V-sign is characterized by a hyperintense signal adjacent to the interpeduncular fossa, which has a sensitivity of 67% in detecting AOP infarction with midbrain involvement.^9,10^ The spectrum of differential diagnoses for bilateral thalamic involvement is vast, including low-grade glioma, metabolic disorders (Wernicke encephalopathy and osmotic myelinolysis), and venous sinus thrombosis, which could delay diagnosis when the clinical history is limited.^9^ In addition, diagnostic challenges associated with AOP strokes primarily stem from small diameter of AOP vessels, which is rarely visualized through MRA (magnetic resonance angiography), computed tomography angiography (CTA), or even conventional angiography. Therefore, the identification of these vessels is not a prerequisite for diagnosis.^10^ However, in rare instances, a thrombus can be detected as a filling defect at the origin of the Percheron artery, as observed in the CTA of our patient (Figure 3). Diagnosing AOP strokes is challenging due to the lack of localizing sequelae, potentially leading to delays in optimal management in acute thrombolysis scenarios.^10^ Therefore, a high index of suspicion of sudden changes in mental status and gaze palsy should warrant timely assessment to prevent unfavorable irreversible outcomes.^9,10^ Prognosis for AOP strokes can vary significantly, with favorable outcomes observed in patients who receive timely treatment.^11^

Saida et al. reported a 61% positive outcome in 18 patients with AOP strokes, as assessed by their ability to resume ADLs (activities of daily living) independently.^11^ The literature indicates that AOP strokes commonly manifest with symptoms such as hypersomnolence, memory deficits, and impaired arousal, which are likely correlated with the impaired pivotal function of the thalami in regulating sleep and consciousness through the polysynaptic network of the ARAS via the adrenergic and dopaminergic pathways.^12^ The thalamus is a complex neuroanatomical structure in its sub-nuclei, which plays a crucial role in facilitating ocular motility and maintaining consciousness.^13^ Furthermore, it serves as a vital relay station for pathways between the cortex and the brainstem, which may explain gaze palsy and hemianopia observed in AOP infarcts.^13^

Anatomically speaking, thalamic anterior intralaminar and paralaminar nuclei, referred to as the “oculomotor thalamus”, play a crucial role in facilitating eye movements through their substantial connections with both the frontal eye field and the supplementary eye field.^5^ Interestingly, in the 1980s, Schlag and Schlag-Rey pioneered in establishing thalamocortical functions in self-paced eye saccades, which explains residual vertical eye movement impairment in our patient, with dorsomedial nuclei being involved among other sub-nuclei in thalamic stroke.^5^ However, our patient initially presented with a sudden onset of bilateral complete loss of vision followed by unresponsiveness that subsequently improved with thrombolysis. In a study conducted by Çetin et al., the clinical and cognitive spectrum of AOP infarction was assessed over a 12-month follow-up.^14^ The findings showed that all 10 patients included in the study experienced cognitive and behavioral impairments across various domains: executive function, processing speed, and working and episodic memory.^14^

The consensus on treating AOP stroke is through secondary preventative measures (antiplatelets and statins) and the identification of underlying thromboembolic causes. Among these, cardioembolic sources tend to be the predominant etiology in reported cases.^11^ One particular case reported an AOP stroke due to APS (anti-phospholipid syndrome), which was treated with dabigatran.^15^ However, treatment with IV thrombolysis in the absence of contraindications is pivotal in acute settings, particularly in light of a high index of suspicion regarding the sudden onset of symptoms, as was the case with our patient. We found only three reported cases of AOP stroke treated with IV thrombolysis in the literature.^8,16,17^ Our patient had a partial filling defect in the left P1 segment on CTA, which was managed with thrombolysis without the need for thrombectomy. However, thrombectomy of the affected P1 segment should be considered in cases of complete occlusion. The patient was subsequently managed by a multidisciplinary approach that included the administration of antiplatelets and statin, as well as intensive cognitive, physical rehabilitation, and speech therapy.

## Conclusion

AOP is a rare variant of thalamic blood supply, and its occlusion usually results in a rare bilateral thalamic stroke. This type of stroke remains a radiological diagnosis due to the absence of specific localizing neurological signs. Therefore, a high index of suspicion is pivotal for timely diagnosis and optimal management with thrombolysis to prevent AOP sequelae in patients who present with sudden onset loss of consciousness with no other apparent etiology.

### List of abbreviations

[Table tbl2]

### Ethics approval and consent to participate

This case report was approved by the Hamad Medical Corporation's Medical Research Center [MRC 04-23-476].

### Consent for publication

Written informed consent was obtained from the patient for the publication of this case report, along with any accompanying images and photographs.

### Funding

The open access publication fees for this case report were funded by the Qatar National Library.

### Competing interests

The authors have no conflicts of interest to disclose.

### Authors' contribution

**AS:** Writing the initial draft of the manuscript, medical management of the case, revising the manuscript critically, and literature review. **IE:** Medical management of the case, revising the manuscript critically, and literature review. **KZ:** Revising the manuscript critically and literature review. **SH:** Conceptualization and supervision, medical management, revising the manuscript critically, and literature review. **AM, KH:** Conceptualization and supervision, medical management. **MM, OK:** Conceptualization, management and supervision, obtaining radiological images.

## Figures and Tables

**Table 1.** Description of thalamic arterial supply variants

Types	Anatomical description	Prevalence (%)

I	Bilateral paramedian thalamic with midbrain	43

IIA	Bilateral paramedian thalamic without midbrain	38

IIB	Bilateral paramedian thalamic with anterior thalamus and midbrain	14

III	Bilateral paramedian thalamic with anterior thalamus without midbrain	5


**Table tbl2:** 

ADLs	Activities of Daily Living

AOP	Artery of Percheron

ASA	Aspirin

CT	Computed Tomography

CTA	Computed Tomography Angiography

ED	Emergency Department

FLAIR	Fluid-Attenuated Inversion Recovery

IV	Intravenous

MRA	Magnetic Resonance Angiography

MRI	Magnetic Resonance Imaging

